# What socio-demographic characteristics of university students in Southern Germany predict their urban nature connectedness?

**DOI:** 10.1371/journal.pone.0272344

**Published:** 2022-08-03

**Authors:** Dorothea M. I. Schönbach, Ximena Tiscareno-Osorno, Tadhg E. MacIntyre, Stephen Smith, Deirdre MacIntyre, Yolanda Demetriou

**Affiliations:** 1 Department of Sport and Health Sciences, Technical University of Munich, Munich, Germany; 2 All Institute, Department of Psychology, Maynooth University, Maynooth, Co. Kildare, Ireland; 3 Institute of Child Education and Psychology, Europe, Maynooth, Co. Kildare, Ireland; LSU Health Sciences Center New Orleans: Louisiana State University Health Sciences Center, UNITED STATES

## Abstract

Promoting mental health addresses a global societal challenge. Nature connectedness, or relatedness to natural systems, is associated with increased well-being and mental health. Among urban populations, nature connectedness has been reported as lower. Nature connectedness in urban settings has been somewhat overlooked by researchers. This cross-sectional online study addressed this issue by identifying socio-demographic predictors of urban nature connectedness among 165 students, aged 20 to 40, from the Technical University of Munich. Analysis of the data from 153 female university students was conducted using ordinal regressions, (a) separately and (b) merged with their 12 male counterparts. A separate gender analysis for males could not be performed, due to sample size limitations. Although access to nature and a considerable nature dose in terms of frequency and duration of nature contact were noted, possibly achieved through the engagement in outdoor activities, urban nature connectedness was rather low. Results showed that urban nature connectedness was negatively predicted by living in a rural area and positively predicted by engagement in outdoor activities and increased hours per week spent in nature. This information can help researchers operationalize the construct of urban nature connectedness. Furthermore, it can aid the development of interventions aimed at promoting urban nature connectedness, by encouraging urban inhabitants to (repeatedly) get in contact with and spend time in provided high-quality urban nature (e.g., during outdoor activities). To draw conclusions about causality, further research is required to identify a clear cause-effect relationship between socio-demographic characteristics and urban nature connectedness. Based on a researched dose-response relationship, a(n) (inter)national recommendation for the duration of nature contact should be established to promote urban nature connectedness and, therewith, health in urban inhabitants. Future research should also investigate further potential individual influencing factors, as well as gender and within/between-country differences among urban inhabitants.

## Introduction

More than half of the world’s population lives in urban areas [1]. By 2050, this estimate is projected to increase to 68%. Through the establishment of the “Sustainable Development Goals”, the United Nations demands sufficient access to safe and inclusive green spaces in urban areas by 2030 (target 11.7) [2]. In 2017, the World Health Organization (WHO) highlighted the importance of urban green spaces by recommending that, as a rule of thumb, access to a minimum of 0.5–1 hectare of green space should be provided within 300 meters of the home of every urban inhabitant, as the crow flies (i.e., approximately a walk of 5 minutes) [3]. However, more than half of urban inhabitants (i.e., > 60%) live in areas where the availability of green space is insufficient [4].

Findings suggest that the ratio of urban green space improves mental health (i.e., stress, depression) [5]. Nature dose (i.e., frequency and duration), which negatively relates to the level of urbanization [6], positively impacts depression [6, 7], high blood pressure [7], social cohesion [6, 7], self-reported health [6], and physical activity [6, 7]. Nature connectedness, found to be lower in urban areas [8], is also related to psychological health, according to the “biophilia hypothesis” [9]. Previous research supports this hypothesis for eudaimonic well-being (i.e., personal growth) [10], hedonic well-being (i.e., vitality, positive affect, life satisfaction) [11], and mental health (i.e., reduction of medical treatment for depression and mental distress) [12]. The promotion of positive mental health is of global importance, as approximately 11% of the world’s population (i.e., one in ten people) is affected by mental health problems globally [13]. However, as increasing proportions of the population live in urban areas with limited access to nature and limited nature dose coupled with lower nature connectedness, they may be denied mental health benefits.

Given that individual differences have been consistently found in nature connectedness [14], it is worthwhile to attempt to identify the reasons underlying this variability (e.g., living in urban areas). Several influencing factors have been identified at the individual level in previous studies conducted across different countries (Table 1). For nature connectedness among urban inhabitants, we found only one study using an Australian sample. They reported a positive correlation between nature connectedness and both previous experiences and duration of current nature experiences [15].

**Table 1:** Influencing factors of nature connectedness at the individual level.

Variables	Countries: Results
Age	• USA/Russia: Positive correlation with age [16] • UK: Differences between age groups [17, 18]
Gender	• Peru, UK, China, and Germany: Differences in favor of females [16–20] • Ecuador: Differences in favor of males [20]
Nationality	• Germany/Ecuador: Differences in favor of Ecuadorians [20]
Attendance of a green school	• China: Positive correlation [19]
Educational level	• Peru, USA, and China: Positive correlation [16, 19] (e.g., in favor of college and graduate degrees [21])
Course of studies	• USA: Differences in favor of environmental issues [21]
Employment status	• UK: Differences to the disadvantage of being in education compared with being in a full-time position, unemployed, part-time position, and retired [17]
Makeup of residential area during childhood	• Switzerland: Differences in favor of rural areas [16]
Near-home natural environment	• USA: Positive correlation [22]
Previous nature experiences/exposures	• USA, Brazil, UK, Canada, and Sweden: Positive correlation [22–24] (e.g., pleasant, greater, direct [25] and frequent [26] contact, green exercise [23], extent of collecting behavior [27, 28])
Current and pleasant nature contact	• USA, Brazil, France, Austria, Canada/New Zealand/USA, Singapore, Australia, and Germany/Ecuador: Positive correlation [26, 29], including frequency [14, 26, 29–32] of time [14, 20, 31–33] spent in recreation [34] or activities [14, 30, 31] for leisure [29] in nature or outdoors [14, 31] (e.g., horseback riding and dog [35]/pet [14] ownership)
Time spent per day using smartphones	• Mixed sample (i.e., UK, USA, Australia, Canada, other countries): Negative correlation [36]
Travelling	• China: Positive correlation [19]

e.g. = for example; i.e. = that is.

Based on our review of the extant literature on nature connectedness, several gaps can be identified. To bridge these gaps, further research should (a) investigate nature connectedness in a European context, and in terms of between-country differences (e.g., to connect with cultural differences) and (b) explore nature connectedness at the individual level in urban settings, and in terms of within- as well as between-country differences. This study seeks to address research gap (b), by identifying socio-demographic predictors of urban nature connectedness in a sample of university students in Southern Germany. We establish the construct of urban nature connectedness, which will be operationalized through our research, because nature in the urban context has specific characteristics (e.g., traffic noise, rare outdoor experience of being alone, blue and green spaces that are well-tended, limited biodiversity) that appear to be insufficiently considered in the current construct of nature connectedness and related measuring instruments [37].

## Methods

### Participants

In June 2021, 190 university students, enrolled in the mandatory module “Health promotion programs” in the sixth semester of the Bachelor course of studies “Health Science” at the Technical University of Munich, were invited to volunteer to participate in this study.

### Data collection

Collection of cross-sectional data took place online, using Unipark, and was supervised face-to-face, using Zoom, by an experienced researcher (DMIS) and five previously trained assistants (LL, SM, AE, PW, JB/DS). Due to the large number of university students, the course cohort was divided in two groups. In two consecutive weeks during the semester, one group at a time filled out a survey during the 90 minutes class. Furthermore, breakout sessions were availed of to further divide each group per week into smaller groups, in which participants were separated for gender. To establish an atmosphere of trust and avoid mixing gender-specific perspectives, female university students were supervised by a female researcher or assistant, and their male counterparts by a male assistant. This procedure was chosen as this study was the first part of a larger investigation including a qualitative data collection. Before accessing the survey, participants were required to read and sign an online informed consent form. University students were then asked the following questions about their socio-demographic characteristics: (a) age, (b) gender, (c) zip code of residential area, (d) marital status, (e) parental status, (f) extent of media use, (g) religious affiliation, (h) dog ownership, (i) access to nature, (j) time (in days and hours per week) spent in nature, (k) engagement in outdoor activities, (l) self-reported urban nature connectedness on a four-point Likert scale ranging from 1 = not at all, 2 = rather not, 3 = rather yes, 4 = very much. Questions i-k were not restricted to a certain urbanization level. As the course attendance was not compulsory, university students received additional course credit in exchange for their participation. Therefore, data collection was not anonymous but accessed data was fully anonymized. According to the German Research Foundation, a statement by an ethics committee was not required for this kind of study as we informed participants about the aim of the study and this study did not involve vulnerable groups, a high level of emotions/stress, social/physical risks, or deception [38].

### Analysis

Ordinal regressions, using IBM SPSS Statistics V.27, were performed for 153 eligible female university students (a) separately, in line with the demand from previous research [39], and (b) merged with their 12 eligible male counterparts (S1 Dataset). As the participation quota of male university students was too small, no separate gender analysis for them was performed. The analyses were aimed at predicting university students’ probability of being in a higher, as opposed to a lower, category of urban nature connectedness (dependent variable), based on their socio-demographic characteristics (independent variables). Each of the following independent variables, classified as metric data, was considered as covariate: (a) age, (b) extent of media use, (c) time (days and hours) spent in nature. Each of the following independent variables, classified as nominal data, was considered as factor: (d) gender, (e) residential area based on zip code (i.e., rural: 2000–5000 inhabitants, small town: 5000–20,000 inhabitants, medium-sized town: 20,000–100,000 inhabitants, city: >100,000 inhabitants), (f) marital status, (g) parental status, (h) religious affiliation, (i) dog ownership, (j) access to nature, (k) engagement in outdoor activities. By default, IBM SPSS Statistics V.27 treats the highest coded category of nominal data as the reference value. We defined the threshold for significance as follows: *p* ≤ 0.05.

## Results

The 165 eligible participants were aged between 20 and 40 years old. Independent of gender, most participating university students lived in the city, followed by medium-sized town, small town, and rural area (S1 Table). The majority of female and male university students had access to nature (98.7% versus 100%) and engaged in outdoor activities (87.6% versus 91.7%). Both genders spent a similar number of days (female: 4.5 ± 1.8, male: 4.9 ± 1.6) and hours (female: 14.3 ± 10.6, male: 14.4 ± 8.9) per week in nature. The urban nature connectedness of the sample was identified as rather low (female: 2.8 ± .7, male: 2.8 ± .4).

For the separate gender analysis of female university students, ordinal regressions revealed the following significant predictors of urban nature connectedness (S2 Table): (a) Living in a rural area predicted a decrease of urban nature connectedness (*p* < .001; Estimate = -1.778 [CI 95: -2.820, -.736]). (b) Engagement in outdoor activities (i.e., going for a walk, jogging, riding a (mountain) bike, hiking, climbing, bouldering, horseback riding, surfing, diving) predicted an increase of urban nature connectedness (*p* = .006; Estimate = 1.305 [CI 95: .369, 2.242]).

For the merged analysis of female and male university students, ordinal regressions revealed the following additional significant predictor of urban nature connectedness: (c) Increased time spent in nature (in hours per week) predicted an increase of urban nature connectedness (*p* = .043; Estimate = .031 [CI 95: .001, .062]).

## Discussion

The aim of this study, conducted in Southern Germany, was to predict university students’ urban nature connectedness, based on their socio-demographic characteristics. In general, gender differences did not consistently emerge, which is not surprising given that gender was not balanced across the sample. More than half of participating female university students and three-quarters of their male counterparts lived in the city, almost all had access to nature and engaged in outdoor activities that require physical activity, and participants spent, on average, more than half of the days of the week and approximately 9% of their weekly hours in nature. However, results indicated that participants were rather disconnected with urban nature based on self-report. Analyses also showed that urban nature connectedness was found to decrease in university students who lived in a rural area. In contrast, engaging in outdoor activities and spending more time in nature (in hours per week) was associated with an increase of urban nature connectedness.

### Urban nature connectedness

As Munich’s percentage of green area (i.e., surface of green spaces) of 25% was found to be exactly in line with the WHO recommendation in the ISGlobal ranking of cities [4], it is not surprising that most university students had access to nature. Despite the extent of this access, coupled with a considerable nature dose (i.e., frequency in days and duration in hours per week), which was possibly gained through the engagement in outdoor activities, they rather reported a lack of urban nature connectedness. Low nature connectedness among urban inhabitants is consistent with previous research [8]. However, as the distribution of the urbanization level of residential areas within the sample indicated that 42.9% of university students were not living in the city, which predicted a decrease of urban nature connectedness, the average value of urban nature connectedness might be biased. When referring to research on nature connectedness across the lifespan, findings suggest that it dips among adolescents and young adults [17, 18], which might also have biased the result of falling into the low category among our respondents. Another explanation for the low urban nature connectedness could be (a) directly or (b) indirectly related to nature dose. Rather than frequency, the duration of spending time in nature was found to be a significant positive predictor of urban nature connectedness, which is again in line with previous research [15]. The sample substantially exceeded the health and well-being recommendation of spending at least 2 hours per week in nature [40] and at least 30 minutes in urban nature [7], perhaps owing somewhat to their recruitment from the “Health Science” course of studies within the university. This affiliation may have enhanced the likelihood of the sample being well educated about, and more conscious of, the various nature-related health benefits, as well as being more committed to health-promoting strategies. (a) The reported duration of approximately 14 hours per week still might not have been enough to fall into a category considered as high urban nature connectedness, possibly because nature connectedness requires time, particularly in urban areas. This explanation suggests that urban nature connectedness may be more a state rather than a trait, which raises questions as to how many hours per week are needed in order to connect with urban nature and what measures can be taken to ensure urban inhabitants meet this requirement. Living in an industrialized society, surrounded by technology and urbanization, may account for a notable proportion of the increased time spent indoors (e.g., approximately 65% at home) [41, 42] and the reduction in time spent in urban nature. (b) The quality of contact with urban nature (e.g., in terms of intensity) might have been subjectively perceived as insufficient by university students and therefore, might have hindered the establishment of a meaningful connectedness with urban nature. An indication for this suggested hypothesis could relate to Munich’s normalized difference vegetation index (i.e., greenness) of 0.522, which fails to meet the WHO recommendation of 0.545, according to the ISGlobal ranking of cities [4]. This means that the quality of urban nature in the city could be of more variable or limited quality (i.e., built environment in the foreground and mundane nature in the background), when compared with the more pure and “natural” nature which may be readily available in rural areas (i.e., wild or untrammeled nature in the foreground and built environment in the background). Here, questions arise as to what needs have to be considered for urban nature connectedness. In addition to this publication, we will soon provide an in-depth needs assessment, based on concept mapping, which is expected to help to provide some answers in line with the demand from previous research [43] and will form a bedrock of the development process of a relevant follow-on intervention in this research field [44]. Concept mapping is a mixed methods approach [45] that combines quantitative and qualitative research [46], as demanded from previous research [43], and gives deep insights into a predefined research question directly answered from the target group in the field. For now, providing urban inhabitants with high-quality urban nature (e.g., high levels of biodiversity and low levels of co-hazards of noise and air pollution), in line with the WHO’s global action plan on physical activity 2018–2030 [47], might enhance the level of encouragement and be a greater reason to engage with urban nature, perhaps thereby promoting an elevation of urban nature connectedness. Such reasons could include engagement in outdoor activities, which can, but do not necessarily have to, require being physically active (e.g., taking photos in a woodland, fishing by a river), and urban nature experiences/exposure (e.g., bathe in the sun by a lake, working from home in a park).

### Strengths and limitations

This study is unique, to the best of our knowledge, in examining the socio-demographic predictors of urban nature connectedness. It was inspired by a broad range of predictors of nature connectedness identified in previous research, our own suggestions (i.e., marital and parental status), and demands for research from previous studies (i.e., religious affiliation [48], residential area [16]). As opposed to predefining the construct of urban nature connectedness and its dimensions, this study sought to have university students shape and rate this construct, according to their own self-defined understanding. The approach was adopted due to the existing lack of clarity regarding the operationalization of this construct, which this study sought to address and ameliorate. Based on results from previous research, wherein a central tendency bias emerged as a significant factor in the study outcomes, university students were strongly encouraged to take a firm stand on their connectedness with urban nature within the survey, so as to minimize the impact of this biasing effect. Additionally, the completion of a separate analysis for female university students represents a further strength of this study, as it provides valuable insights into the diversity dimension “gender” and identifies potential gender-related differences/inequalities.

However, a generalization of the study results is limited due to the geographical restriction of collecting data in Southern Germany, and the small sample size included. The sample is non-representative for the general population as it involved a convenience sample of university students, primarily due to their availability, accessibility, and affection of mental health issues and impaired well-being [49] during the Covid-19 pandemic. To avoid a recall bias, no information regarding previous urban nature experiences was requested. During data collection, it was noted that the question concerning “extent of media use (in hours per day)”, which included the use of smartphone, TV, computer, tablet etc., was a cause of uncertainty. Owing to the increased reliance on distance learning during the Covid-19 pandemic, participants felt that this question was not formulated in a sufficiently clear and unambiguous manner, as to whether or not hours spent engaging with media for the purpose of engaging with, and completing, coursework should be included in the answer. Additionally, further issues were identified regarding the self-reported urban nature connectedness rating, as no prior consideration was given of seasonal dependence and weather conditions. An unequal distribution between genders, caused by an over-representation of female university students in the overall sample that reflects the gender ratio in the Bachelor course of studies “Health Science” at the Technical University of Munich, precluded the completion of a separate gender analysis for their male counterparts as the sample size for male participants was too small. Lastly, this study gives an overview of predictors, as opposed to determinants, of urban nature connectedness and, as such, we cannot draw conclusions about causality (e.g., it remains unclear if more time spent in nature led to urban nature connectedness or vice versa).

## Conclusions

This study provides new insights into urban nature connectedness and its socio-demographic predictors in Germany. Although conclusions can only be drawn with caution, the study results can help researchers operationalize the construct of urban nature connectedness. It was found that urban nature connectedness was rather low among university students in Southern Germany, despite most of the sample having ample access to nature in line with the WHO recommendation, engaging in outdoor activities, and receiving a considerable nature dose. Socio-demographic characteristics that predicted urban nature connectedness were: (-) living in a rural area, (+) increased weekly hours spent in nature and engagement in outdoor activities. These results suggest, in order to promote urban nature connectedness, interventions need to be developed aimed at providing high-quality urban nature, which indicators have to be defined first, that encourage urban inhabitants to (repeatedly) get in contact with and spend time in it (e.g., during outdoor activities).

The challenge of future research lies in identifying socio-demographic determinants of urban nature connectedness in a representative (i.e., more heterogeneous) sample of the general population, including a balanced distribution between genders to examine potential gender differences. Only determinants provide a clear cause-effect relationship and reveal the socio-demographic characteristics that can cause an increase and maintenance of urban nature connectedness. Further research is warranted to confirm the construct of urban nature connectedness as either a temporary state or stable trait, with a view to establishing a(n) (inter)national recommendation on how to promote urban nature connectedness and therewith health in urban inhabitants based on a dose-response relationship. As the aim of this study was not to give a complete but comprehensive picture of predictors on urban nature connectedness, future research should examine further potential influencing factors at the individual level (e.g., socio-economic status including educational level, school attended, employment status, income). Finally, it is recommended that future studies conduct research exclusively in urban inhabitants to gain a clearer picture of factors influencing urban nature connectedness and also consider the impact of different cultural contexts, so as to examine potential invariances within/between countries.

## Supporting information

S1 Table Socio-demographic characteristics of university students. *n* = sub-sample; yrs. = years.
(DOCX)

S2 Table Ordinal regressions of university students’ socio-demographic characteristics and urban nature connectedness. CI = confidence interval; *N* = overall sample, *n* = sub-sample; *p* = probability value; ref. = reference value; yrs. = years.
(DOCX)

S1 Dataset (XLSX)

## References

[1] un.org [Internet]. New York: United Nations; c2018 [cited 2022 Mar 14]. Available from: https://www.un.org/development/desa/en/news/population/2018-revision-of-world-urbanization-prospects.html

[2] sdgs.un.org [Internet]. New York: United Nations; c no date [cited 2022 Mar 14]. Available from: https://sdgs.un.org/goals

[3] World Health Organization. Urban green spaces: a brief for action [Internet]. Copenhagen: No press; 2017 [cited 2022 Mar 14]. Available from: https://www.euro.who.int/__data/assets/pdf_file/0010/342289/Urban-Green-Spaces_EN_WHO_web3.pdf

[4] isglobalranking.org [Internet]. Barcelona: ISGlobal; c no date [cited 2022 Mar 14]. Available from https://isglobalranking.org/

[5] Lee HJ, Lee DK. Do sociodemographic factors and urban green space affect mental health outcomes among the urban elderly population?. Int J Environ Res Public Health. 2019 Mar 04;16(5):789. doi: 10.3390/ijerph16050789 30836691 PMC6427606

[6] Cox DTC, Shanahan DF, Hudson HL, Fuller RA, Gaston KJ. The impact of urbanisation on nature dose and the implications for human health. Landsc Urban Plan. 2018;179:72–80.

[7] Shanahan DF, Bush R, Gaston KJ, Lin BB, Dean J, Barber E, et al. Health benefits from nature experiences depend on dose. Sci Rep. 2016;6:28551. doi: 10.1038/srep28551 27334040 PMC4917833

[8] Braito MT, Böck K, Flint C, Muhar A, Muhar S, Penker M. Human-nature relationships and linkages to environmental behaviour. Environ Values. 2017;26(3):365–89.

[9] Wilson EO. Biophilia. Cambridge, MA: Harvard University Press; 1984.

[10] Pritchard A, Richardson M, Sheffield D, McEwan K. The relationship between nature connectedness and eudaimonic well-being: a meta-analysis. J Happiness Stud. 2019; 21:1145–67.

[11] Capaldi CA, Dopko RL, Zelenski JM. The relationship between nature connectedness and happiness: a meta-analysis. Front Psychol. 2014;5:976. doi: 10.3389/fpsyg.2014.00976 25249992 PMC4157607

[12] White MP, Elliott LR, Grellier J, Economou T, Bell S, Bratman GN, et al. Associations between green/blue spaces and mental health across 18 countries. Sci Rep. 2021 Apr 26;11(1):8903. doi: 10.1038/s41598-021-87675-0 33903601 PMC8076244

[13] Dattani S, Ritchie H, Roser M [Internet]. England/Wales (UK): Global Change Data Lab. c2018 - [cited 2022 Mar 14]. Available from: https://ourworldindata.org/mental-health

[14] Nisbet EK, Zelenski JM, Murphy SA. The nature relatedness scale: linking individuals’ connection with nature to environmental concern and behavior. Environ Behav. 2008 Aug 01;41(5):715–40.

[15] Cleary A, Fielding KS, Murray Z, Roiko A. Predictors of nature connection among urban residents: assessing the role of childhood and adult nature experiences. Environ Behav. 2018 Nov 12;52(6):579–610.

[16] Clayton S, Czellar S, Nartova-Bochaver S, Skibins JC, Salazar G, Tseng Y-C, et al. Cross-cultural validation of a revised environmental identity scale. Sustainability. 2021 Feb 23;13(4):2387.

[17] Richardson M, Hunt A, Hinds J, Bragg R, Fido D, Petronzi D, et al. A measure of nature connectedness for children and adults: validation, performance, and insights. Sustainability. 2019 June 12;11(12):3250.

[18] Hughes J, Rogerson M, Barton J, Bragg R. Age and connection to nature: when is engagement critical?. Front Ecol Environ. 2019 Apr 01;17(5):265–9.

[19] Li W, Lang G. Effects of green school and parents on children’s perceptions of human-nature relationships in China. Child Ind Res. 2014 Aug 28;8(3):587–604.

[20] Dornhoff M, Sothmann J-N, Fiebelkorn F, Menzel S. Nature relatedness and environmental concern of young people in Ecuador and Germany. Front Psychol. 2019;10:453. doi: 10.3389/fpsyg.2019.00453 30899233 PMC6416211

[21] Mayer FS, McPherson Frantz C. The connectedness to nature scale: a measure of individuals’ feeling in community with nature. J Environ Psychol. 2004;24(4):503–15.

[22] Cheng JC-H, Monroe MC. Connection to nature: children’s affective attitude toward nature. Environ Behav. 2010 Nov 07;44(1):31–49.

[23] Wood CJ, Smyth N. The health impact of nature exposure and green exercise across the life course: a pilot study. Int J Environ Health Res. 2019 Mar 21;30(2):226–35. doi: 10.1080/09603123.2019.1593327 30895804

[24] Windhorst E, Williams A. Growing up, naturally: the mental health legacy of early nature affiliation. Ecopsychology. 2015 Sep 28;7(3):115–25.

[25] Rosa CD, Cabicieri Profice C, Collado S. Nature experiences and adults’ self-reported pro-environmental behaviors: the role of connectedness to nature and childhood nature experiences. Front Psychol. 2018;9:1055. doi: 10.3389/fpsyg.2018.01055 30013494 PMC6036283

[26] Tam K-P. Concepts and measures related to connection to nature: similarities and differences. J Environ Psychol. 2013;34:64–78.

[27] Berry TH, Lekies KS. Childhood collecting in nature: quality experience in important places. Child Geogr. 2018 Apr 12;17(1):118–31.

[28] Lekies KS, Berry TH,. Everyone needs a rock: collecting items from nature in childhood. Child Youth Environ. 2013;23(3):66–88.

[29] Chew PKH. Psychometric evaluation of two instruments to assess connection to nature. Psyecology. 2019 May 10;10(3):313–43.

[30] Navarro O, Olivos P, Fleury-Bahi G. “Connectedness to nature scale”: validity and reliability in the French context. Front Psychol. 2017;8:2180. doi: 10.3389/fpsyg.2017.02180 29312052 PMC5733057

[31] Nisbet EK, Zelenski JM. The NR-6: a new brief measure of nature relatedness. Front Psychol. 2013;4:813. doi: 10.3389/fpsyg.2013.00813 24198806 PMC3814587

[32] Meis-Harris J, Borg K, Jorgensen BS. The construct validity of the multidimensional AIMES connection to nature scale: measuring human relationships with nature. J Environ Manage. 2021;280:111695. doi: 10.1016/j.jenvman.2020.111695 33298399

[33] Hatty MA, Smith LDG, Goodwin D, Mavondo FT. The CN-12: a brief, multidimensional connection with nature instrument. Front Psychol. 2020;11:1566. doi: 10.3389/fpsyg.2020.01566 32760325 PMC7372083

[34] Rosa CD, Collado S, Cabicieri Profice C, Pires PP. The 7-items version of the connectedness to nature scale: a study of its validity and reliability with Brazilians. Curr Psychol. 2020:1–6.

[35] Schwarzmüller-Erber G, Stummer H, Maier M, Kundi M. Nature relatedness of recreational horseback riders and its association with mood and wellbeing. Int J Environ Res Public Health. 2020 June 10;17(11):4136. doi: 10.3390/ijerph17114136 32531937 PMC7312614

[36] Richardson M, Hussain Z, Griffiths MD. Problematic smartphone use, nature connectedness, and anxiety. J Behav Addict. 2018 Feb 12;7(1):109–16. doi: 10.1556/2006.7.2018.10 29415553 PMC6035021

[37] Sobko T, Jia Z, Brown G. Measuring connectedness to nature in preschool children in an urban setting and its relation to psychological functioning. PLoS One. 2018 Nov 29;13(11):e0207057. doi: 10.1371/journal.pone.0207057 30496300 PMC6264829

[38] dfg.de [Internet]. Bonn: Deutsche Forschungsgemeinschaft.; c2022 [cited 2022 Mar 18]. Available from: https://www.dfg.de/en/research_funding/faq/faq_humanities_social_science/index.html

[39] Shapiro JR, Klein SL, Morgan R. Stop ’controlling’ for sex and gender in global health research. BMJ Glob Health. 2021;6(4):e005714. doi: 10.1136/bmjgh-2021-005714 33846145 PMC8048018

[40] White MP, Alcock I, Grellier J, Wheeler BW, Hartig T, Warber SL, et al. Spending at least 120 minutes a week in nature is associated with good health and wellbeing. Sci Rep. 2019 June 13;9(1):7730. doi: 10.1038/s41598-019-44097-3 31197192 PMC6565732

[41] Leech JA, Nelson WC, Burnett RT, Aaron S, Raizenne ME. It’s about time: a comparison of Canadian and American time-activity patterns. J Expo Anal Environ Epidemiol. 2002;12(6):427–32. doi: 10.1038/sj.jea.7500244 12415491

[42] Brasche S, Bischof W. Daily time spent indoors in German homes–Baseline data for the assessment of indoor exposure of German occupants. Int J Hyg Environ Health. 2005;208(4):247–53. doi: 10.1016/j.ijheh.2005.03.003 16078638

[43] MacIntyre TE, Calogiuri G, Donnelly AA, Warrington G, Beckmann J, Lahart I, et al. Societal challenges, methodological issues and transdisciplinary approaches. In: Donnelly AA, MacIntyre TE, editors. Physical activity in natural settings. Green and blue exercise. London: Routledge; 2020. p. 15–35.

[44] Trochim WMK. An introduction to concept mapping for planning and evaluation. Eval Program Plann. 1989;12(1):1–16.

[45] Trochim W, Kane M. Concept mapping: an introduction to structured conceptualization in health care. Int J Qual Health Care. 2005 May 04;17(3):187–91. doi: 10.1093/intqhc/mzi038 15872026

[46] Burke JG O’Campo P, Peak GL, Gielen AC, McDonnell KA, Trochim WMK. An introduction to concept mapping as a participatory public health research method. Qual Health Res. 2005 Dec 01;15(10):1392–410. doi: 10.1177/1049732305278876 16263919

[47] World Health Organization. Global action plan on physical activity 2018–2030. More active people for a healthier world [Internet]. Geneva: No press; 2018 [cited 2022 Mar 15]. Available from: https://apps.who.int/iris/bitstream/handle/10665/272722/9789241514187-eng.pdf

[48] Dutcher DD, Finley JC, Luloff AE, Buttolph Johnson J. Connectivity with nature as a measure of environmental values. Environ Behav. 2007 July 01;39(4):474–93.

[49] Holm-Hadulla RM, Klimov M, Juche T, Möltner A, Herpertz SC. Well-being and mental health of students during the COVID-19 pandemic. Psychopathology. 2021 Sep 24;54(6):291–7. doi: 10.1159/000519366 34564075 PMC8678268

