# Supplementary material for: What socio-demographic characteristics of university students in Southern Germany predict their urban nature connectedness?
Source: PLoS One. 2022 Aug 3;17(8):e0272344. doi: 10.1371/journal.pone.0272344 (PMC9348682; doi:10.1371/journal.pone.0272344)
Supplement: S1 Table — n = sub-sample; yrs. = years. (DOCX) [file pone.0272344.s001.docx]

**S1 Table. Socio-demographic characteristics of university students.** *n* = sub-sample; yrs. = years.

| **Socio-demographic Characteristics** | **Female University Students**  **(*n* = 153)** | | **Male University Students**  **(*n* = 12)** |
| --- | --- | --- | --- |
| Age (yrs.) | 22.7 ± 2.8 | | 24.0 ± 3.1 |
| Residential area  a) rural  b) small town  c) medium-sized town  d) city | 17 (11.3%)  23 (15.2%)  27 (17.9%)  84 (55.6%) | | 1 (8.3%)  1 (8.3%)  1 (8.3%)  9 (75.0%) |
| Marital status  a) single  b) in a relationship  c) married | 71 (46.4%)  78 (51.0%)  4 (2.6%) | | 5 (41.7%)  7 (58.3%)  0 |
| Parental Status  a) yes = have children  b) no = do not have children | 5 (3.3%)  148 (96.7%) | | 0  12 (100%) |
| Extent of media use (in hours per day) | 6.0 ± 2.6 | | 6.7 ± 3.2 |
| Religious affiliation  a) yes  b) no | 76 (49.7%)  77 (50.3%) | | 5 (41.7%)  7 (58.3%) |
| Dog ownership  a) yes  b) no | 16 (10.5%)  137 (89.5%) | | 1 (8.3%)  11 (91.7%) |
| Access to nature  a) yes  b) no | 151 (98.7%)  2 (1.3%) | | 12 (100%)  0 |
| Time spent in nature (in days per week) | 4.5 ± 1.8 | | 4.9 ± 1.6 |
| Time spent in nature (in hours per week) | 14.3 ± 10.6 | | 14.4 ± 8.9 |
| Engagement in outdoor activities  a) yes  b) no | 134 (87.6%)  19 (12.4%) | | 11 (91.7%)  1 (8.3%) |
| Urban nature connectedness  (1 = not at all, 2 = rather not, 3 = rather yes, 4 = very much) | | 2.8 ± .7 | 2.8 ± .4 |
