# Supplementary material for: What socio-demographic characteristics of university students in Southern Germany predict their urban nature connectedness?
Source: PLoS One. 2022 Aug 3;17(8):e0272344. doi: 10.1371/journal.pone.0272344 (PMC9348682; doi:10.1371/journal.pone.0272344)
Supplement: S2 Table — CI = confidence interval; N = overall sample, n = sub-sample; p = probability value; ref. = reference value; yrs. = years. (DOCX) [file pone.0272344.s002.docx]

**S2 Table. Ordinal regressions of university students’ socio-demographic characteristics and urban nature connectedness.** CI = confidence interval; *N* = overall sample, *n* = sub-sample; *p* = probability value; ref. = reference value; yrs. = years.

| **Socio-demographic Characteristics** | **Female University Students**  **(*n* = 153)** | | | | **Female and Male University Students**  **(*N* = 165)** | | | |
| --- | --- | --- | --- | --- | --- | --- | --- | --- |
|  | **Estimate** | ***p*** | **95% CI** | | **Estimate** | ***p*** | **95% CI** | |
|  |  |  | **Lower** | **Upper** |  |  | **Lower** | **Upper** |
| Gender  a) male  b) female (ref.) |  |  |  |  | .191 | .752 | -.994 | 1.377 |
| Age (yrs.) | .013 | .825 | -.099 | .124 | .016 | .765 | -.091 | .124 |
| Residential area  a) rural  b) small town  c) medium-sized town  d) city (ref.) | -1.778  -.898  .405 | **< .001**  .053  .376 | -2.820  -1.808  -.492 | -.736  .013  1.303 | -1.695  -.873  .279 | **< .001**  .055  .535 | -2.700  -1.765  -.603 | -.690  .018  1.161 |
| Marital status  a) single  b) in a relationship  c) married (ref.) | .836  .783 | .394  .422 | -1.084  -1.131 | 2.755  2.697 | .866  .833 | .377  .394 | -1.057  -1.083 | 2.789  2.750 |
| Parental Status  a) yes = have children  b) no = do not have children (ref.) | -.519 | .552 | -2.232 | 1.194 | -.553 | .529 | -2.272 | 1.166 |
| Extent of media use (in hours per day) | -.058 | .344 | -.180 | .063 | -.067 | .256 | -.183 | .049 |
| Religious affiliation  a) yes  b) no (ref.) | -.067 | .835 | -.692 | .559 | 1.315E-16 | 1.000 | -.609 | .609 |
| Dog ownership  a) yes  b) no (ref.) | -.801 | .115 | -1.798 | .196 | -.732 | .140 | -1.704 | .240 |
| Access to nature  a) yes  b) no (ref.) | 2.087 | .129 | -.609 | 4.782 | 2.148 | .119 | -.553 | 4.850 |
| Time spent in nature (in days per week) | .117 | .190 | -.058 | .292 | .137 | .119 | -.035 | .310 |
| Time spent in nature (in hours per week) | .027 | .085 | -.004 | .058 | .031 | **.043** | .001 | .062 |
| Engagement in outdoor activities  a) yes  b) no (ref.) | 1.305 | **.006** | .369 | 2.242 | 1.247 | **.007** | .335 | 2.159 |
